# The influence of menopause age on gynecologic cancer risk: a comprehensive analysis using NHANES data

**DOI:** 10.3389/fonc.2025.1541585

**Published:** 2025-02-11

**Authors:** Yiliminuer Abulajiang, Tao Liu, Ming Wang, Abidan Abulai, Yumei Wu

**Affiliations:** ^1^ Beijing Obstetrics and Gynecology Hospital, Capital Medical University, Beijing Maternal and Child Health Care Hospital, Beijing, China; ^2^ School of Rehabilitation Medicine, Baoding University of Technology, Baoding, China; ^3^ Department of Endocrinology, The First People’s Hospital of Kashi, (The Affiliated Kashi Hospital of Sun Yat-Sen University), Kashi, China

**Keywords:** menopause age, gynecologic cancer risk, personalized cancer screening, NHANES data analysis, risk stratification

## Abstract

**Background:**

Menopause, a natural transition, affects women’s health risks, including gynecologic cancers. Early menopause, linked to lower estrogen, may increase cancer susceptibility. This study analyzed NHANES data from 1999 to 2020 for 8,219 postmenopausal women to explore the relationship between menopausal age and gynecologic cancers. We used regression models and RCS models to assess the risk.

**Methods:**

This study utilized data from the NHANES spanning 1999 to 2020, focusing on 8,219 postmenopausal women selected through stratified sampling. Variables including socioeconomic factors, health behaviors, nutritional status, and medical history were assessed in relation to participants’ menopausal age and gynecologic cancer prevalence. We analyzed the relationship between menopausal age and gynecologic cancers (cervical, ovarian, and uterine) using multiple regression models. Additionally, we employed RCS models to evaluate nonlinear relationships between menopausal age and gynecologic cancer risk.

**Results:**

Our findings indicate a significant inverse association between menopausal age and the risk of gynecologic cancers. After controlling for confounding factors such as age, race, BMI, and lifestyle variables, a later age at menopause was associated with a reduced risk of cervical, ovarian, and uterine cancers. The RCS model revealed a non-linear, low-L-shaped relationship, particularly highlighting increased cancer risks at younger menopausal ages. Subgroup analyses demonstrated consistent results across demographic and lifestyle factors, confirming the robustness of the observed associations.

**Conclusion:**

This study reveals the link between menopausal age and gynecologic cancer prevalence. Early menopause is a significant risk factor for cervical, ovarian, and uterine cancers. Our findings support tailored cancer screening based on menopausal age, potentially improving preventive care for postmenopausal women.

## Introduction

Gynecological malignancies, including endometrial, cervical, and ovarian cancer, etc., are a leading cause of morbidity and mortality among women, and the yearly number of patients is rising (1, 2). According to statistics, over a million new cases are identified annually, which pose a serious threat to global health. Cervical cancer is the most common gynecological malignancy among women under 40, accounting for 50.4% of cases. Although it can be prevented through vaccination and screening, it remains a leading cause of death, particularly in areas with limited healthcare resources. Following cervical cancer is endometrial cancer, which accounts for 24.2% of cases (3). While its incidence is declining in certain regions, it still contributes significantly to the overall burden of gynecological cancers. Ovarian cancer, at 23%, is the deadliest gynecological malignancy. Due to the lack of effective screening methods, approximately 60% of ovarian cancer cases are diagnosed at an advanced stage, which significantly impacts survival rates (4, 5). Gynecological cancers have a profound impact on women’s health and place a significant financial burden on healthcare systems. Their high incidence and mortality rates require management with complex and expensive therapies, which come with several drawbacks, such as treatment-related complications, obesity, social determinants of health, and economic toxicity (6–8).

Menopause is a normal phase of a woman’s life, marked by a drop in estrogen levels and usually happening between the ages of 45 and 55. Early menopause refers to menopause that begins between the ages of 40 and 45. The mechanisms behind early and delayed menopause, as well as their relationship with the risk of ovarian cancer, involve a complex interplay between genetic, hormonal, and environmental factors. Genetic predisposition plays a significant role in determining the age of menopause. Hundreds of single nucleotide polymorphisms related to menopausal age have been identified, many of which are associated with immune and mitochondrial functions as well as DNA repair processes. These genetic factors can influence the risk of ovarian cancer (9). Postmenopausal women have persistently high levels of follicle-stimulating hormone, and the changes in hormones are associated with an increase in the expression of inflammatory cytokines and oxidative stress markers, which may lead to malignant transformation of ovarian tissue (10). Delayed menopause is significantly associated with an increased risk of ovarian cancer, which is due to prolonged exposure to estrogen that promotes the development of ovarian cancer (11). Early menopause and primary ovarian insufficiency (POI) are associated with reduced lifetime exposure to estrogen, which may lower the risk of ovarian cancer (9, 12). After menopause, the risk of cervical cancer in women may be reactivated or persist due to human papillomavirus (HPV) infection. Guidelines recommend that screening can stop at age 65 if adequate prior screening has been conducted. However, many women tend to stop screening too early, which increases their risk of developing cervical cancer (13). The prevalence of high-risk HPV infections in postmenopausal women is quite high, with a noticeable increase in infection rates after the age of 65. This suggests that postmenopausal women, particularly those over 65, may benefit from ongoing screening (14). Delayed menopause is associated with a higher risk of endometrial cancer, as long-term exposure to estrogen without the balancing effect of progesterone increases the likelihood of endometrial hyperplasia and cancer. Early menopause shortens the duration of estrogen exposure, thereby reducing the risk of endometrial cancer. This protective effect is due to a shorter reproductive lifespan and decreased cumulative estrogen exposure (15). Obesity and metabolic syndrome are significant risk factors for endometrial cancer. Obesity increases the risk of endometrial cancer in postmenopausal women through various mechanisms, including elevated aromatase activity (16). Insulin resistance and hyperinsulinemia are commonly found in metabolic syndrome, which further increases the risk of endometrial cancer by enhancing the proliferation of endometrial cells (17–19). Furthermore, genetic factors, such as the expression of specific cancer genes like PKD1, have been identified as causes of the progression of endometrial cancer in postmenopausal women. These genetic markers can help predict disease progression and guide targeted therapy (20). Postmenopausal women with endometrial cancer typically exhibit more aggressive disease characteristics, such as higher tumor grades and increased lymphatic metastasis, which are influenced by genetic and hormonal factors (21). The relationship between menopause age and gynecological malignancies is quite complex and influenced by various factors. Racial and cultural factors can affect both the age of menopause and the risk of cancer. A study on Korean women found that menopausal hormone therapy does not increase the risk of melanoma, but certain therapies reduce the risk of non-melanoma skin cancer (22). This indicates that cultural and genetic factors may play a role in cancer risk, which can vary among populations. Lifestyle factors, such as diabetes, may interact with menopausal age. However, one study found no association between menopausal age and microvascular complications in women with diabetes, suggesting that other health factors may obscure. There may be a nonlinear relationship between menopause age and cancer risk. For instance, an earlier onset of menopause is associated with an increased mortality rate, indicating a complex (23, 24).

This study analyzed data from the National Health and Nutrition Examination Survey (NHANES), a cross-sectional survey covering the United States from 1999 to 2020. A total of 8,219 postmenopausal women were selected using stratified sampling methods, ensuring a representative sample. We evaluated various factors, including socioeconomic characteristics, health behaviors, nutritional status, and medical history, and analyzed the relationships between menopausal age and gynecologic cancer prevalence using multivariable logistic regression models. Additionally, restricted cubic spline (RCS) regression models were applied to examine any nonlinear relationships between menopausal age and gynecologic cancer risk, further uncovering potentially complex associations.

## Methods

### Study design and sample

The NHANES is a nationally representative, cross-sectional survey. The survey used a complex multi-stage probability sample representing the civilian population in all 50 states and the District of Columbia in the United States (25).

**Figure 1:**
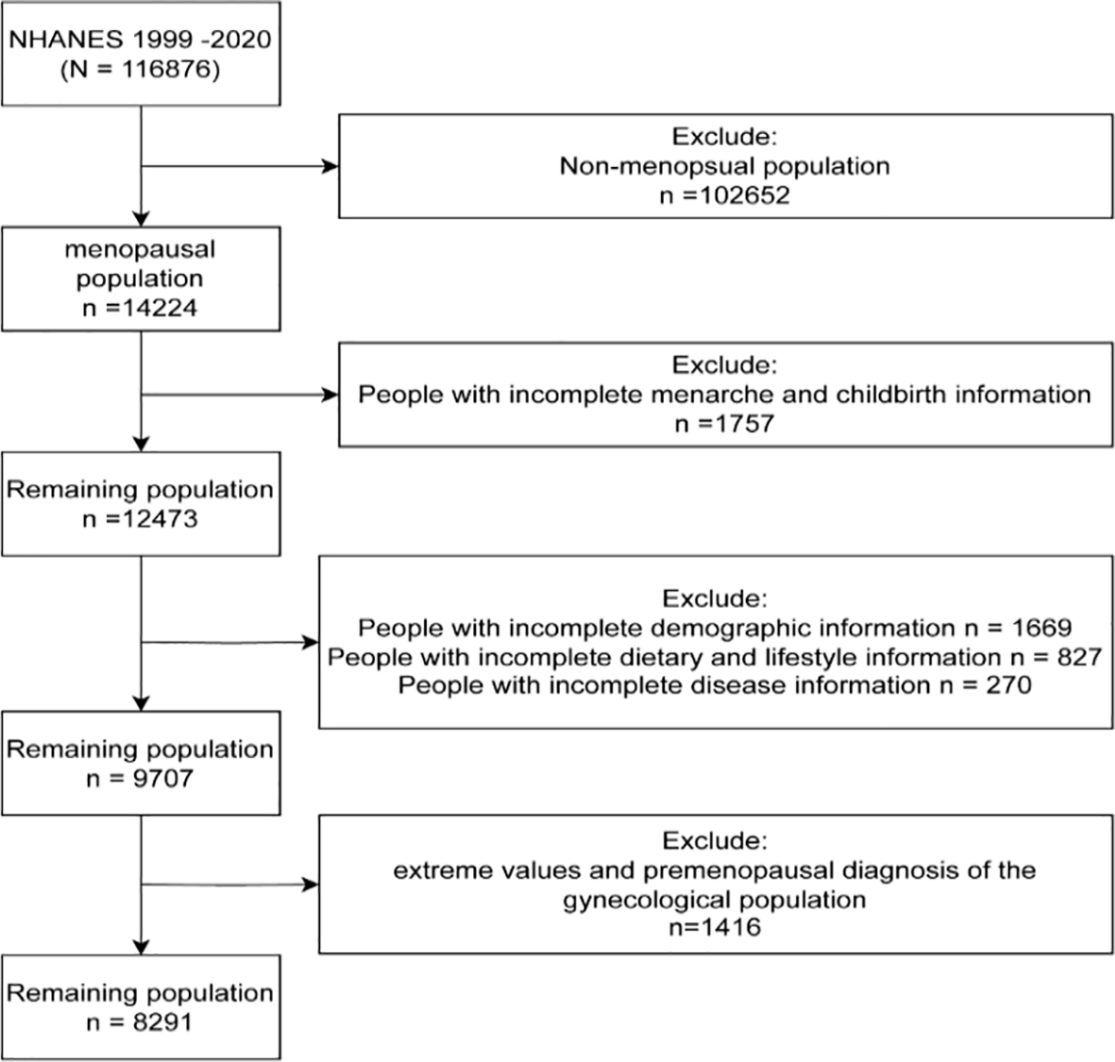
Study design and sample.

We obtained data from the NHANES, which is representative of the civilian population across all 50 states and the District of Columbia. In the current study, a total of 8,291 NHANES participants were included, representing 21,950,882 postmenopausal women over a span of 14 years (1999-2020). The survey received approval from the Institutional Review Board of the National Center for Health Statistics, and all participants provided informed consent. A flowchart illustrating the process of selecting the study sample is shown in **Figure 1**. The total population (n=116,876) was screened, resulting in the identification of premenopausal individuals (n=102,652). Among the postmenopausal women, we excluded those with incomplete information regarding menarche and childbirth (n=1,757). From the remaining 12,473 participants, we further excluded individuals with incomplete demographic, disease, dietary, and necessary testing information (n=2,766). Finally, to enhance the scientific validity and reliability of the results, we excluded individuals with premenopausal gynecological diagnoses (n=1,416) from the remaining population, resulting in our ideal study sample of 8,291 individuals.

### Sociodemographic characteristics

There are several sociodemographic characteristics, including age, race, and educational level. A non-Hispanic white, a non-Hispanic black, a Mexican-American, another Hispanic, and another race can be excluded from the list. In addition to high school, there are levels of education below high school, high school, and higher education.

### Nutritional status

To examine the nutritional quality of the populace, information was gathered from body mass indexes (BMIs) and household property to income ratios (PIRs). Higher PIRs are generally associated with higher levels of physical activity and nutritious intake, as well as higher BMIs compared to low-income populations. The data was divided according to the median, and a cut-off value of 2.3% was chosen as the PIR for households. Screenings were performed on those with BMIs of > 25 kg/m² or ≤ 25 kg/m^2^.

### Habits of behavior

According to how often participants smoked, they were divided into three groups: never smoking, former smoking and now smoking. Alcohol consumption included never drinking, former drinking, mild drinking, moderate drinking, and heavy drinking. In addition to energy intake, behavioral habits are also influenced by population energy intake (kcal).

### A medical condition’s underlying causes

Diabetes mellitus and hypertension were included because they are two diseases associated with women developing gynecologic cancers and progressing to meet their cancer goals. The following are the clinical diagnostic criteria for diabetes mellitus: (1) The doctor makes the diagnosis; (2) a fasting blood glucose level of 7.0 mmol/L; (3) a glycohemoglobin level of greater than 6.5%; (4) a random blood glucose level of 11.1 mmol/L; (5) a two-hour OGTT level of 11.1 mmol/L; (6) any diabetes medications or insulin already being used.

### Statistical analyses

The analyzed data were weighted according to the NCHS. Participants were categorized into two groups based on baseline characteristics according to whether they had gynecologic cancers. Descriptive statistics are used to profile the distribution of participant characteristics, including age, age at menopause, race, education, family PIR, BMI, smoking, alcohol consumption, etc. Data were presented as frequencies with proportions (%), means with standard deviation (SD), or medians with interquartile ranges (IQR). Univariate and multivariate logistic regression analysis between ages at menopause and gynecologic cancers: Crude is an unadjusted model; Model 1 is a model adjusted for age and race; Model 2 is a model adjusted for age, race, first menstruation age, and living birth; Model 3 is a model adjusted for age, race, first menstruation age, living birth, education, BMI, PIR, smoking, alcohol consumption level, energy intake; Model 4 is a model adjusted for age, race, first menstruation age, living birth, education, BMI, PIR, smoking, alcohol consumption level, energy intake, hypertension and diabetes. On this basis, a fully adjusted model was used to assess the association between the age of menopause and major gynecological cancers, including cervical, ovarian, and uterine cancers. To explore the incidence of gynecological cancer in different age groups, participants were further divided into 7 groups based on age at menopause, including ≤30 years, 31-35 years, 36-40 years, 41-45 years, 46-50 years, 51-55 years, and ≥56 years. In the fully adjusted model, a RCS method was used to investigate the non-linear association between age at menopause and gynecologic cancers. In this study, values detected as outliers are treated as missing data and replaced by the result of interpolation. Statistical analyses were conducted using R version 4.4.1 (Posit Software, Boston, MA, USA). A p-value less than 0.05 is considered statistically significant.

## Results

### Baseline characteristics

The weighted baseline characteristics of the participants, which consisted of 8,219 participants grouped by whether they had gynecological cancer, are shown in **Table 1**. The results showed statistically significant differences in gynecologic cancer prevalence by family PIR, smoking, menopause, and first menstruation (*P*<0.05). Compared with participants without gynecological cancer, participants with gynecological cancer had lower Family PIR, more smoking, lower age at menopause, and younger age at first menstruation.

**Table 1:** Participant characteristics (N = 8,219) in NHANES 1999–2020.

Characteristic	Non-gynecological cancer (N = 8,017)	Gynecological cancer (N = 274)	*P* value
Age, years	60.00 (53.00, 69.00)	61.00 (49.00, 70.00)	0.430
Race, %			**0.032**
Non-Hispanic White	3,681.00 (73.54)	173.00 (82.70)	
Non-Hispanic Black	1,801.00 (10.90)	36.00 (5.43)	
Mexican American	1,233.00 (4.96)	32.00 (3.52)	
Other Hispanic	741.00 (4.79)	21.00 (3.82)	
Other Race	561.00 (5.81)	12.00 (4.53)	
Education level, %			0.700
Less than high school	2,261.00 (17.49)	82.00 (19.89)	
High school	2,069.00 (28.36)	74.00 (27.81)	
College or above	3,687.00 (54.15)	118.00 (52.30)	
Family PIR	3.03 (1.58, 5.00)	2.48 (1.24, 3.85)	**0.008**
BMI, kg/m^2^	28.74 (24.76, 33.46)	30.10 (25.60, 34.99)	0.054
Smoke behavior, %			**0.001**
Never	4,812.00 (57.24)	125.00 (42.91)	
Former	1,940.00 (25.51)	75.00 (29.62)	
Now	1,265.00 (17.24)	74.00 (27.48)	
Alcohol consumption, %			0.068
Never	1,800.00 (16.55)	47.00 (10.00)	
Former	1,606.00 (16.80)	68.00 (20.36)	
Mild	2,539.00 (36.58)	86.00 (32.45)	
Moderate	1,308.00 (20.12)	40.00 (21.83)	
Heavy	764.00 (9.95)	33.00 (15.36)	
Energy intake, kcal	1,627.00 (1,260.00, 2,069.00)	1,602.00 (1,215.00, 2,067.00)	0.700
Hypertension, %	2,503.00 (26.09)	80.00 (22.10)	0.190
Diabetes, %	2,006.00 (19.46)	77.00 (19.91)	0.880
Menopause, years	46.00 (39.00, 51.00)	36.00 (30.00, 46.00)	**<0.001**
First menstruation, years	13.00 (12.00, 14.00)	12.00 (11.00, 13.00)	**0.028**
Living birth	2.00 (2.00, 3.00)	2.00 (2.00, 3.00)	0.950

Bold indicates *P* value < 0.05.

### Relationship between the age of menopause and the prevalence of gynecological cancers

The results of univariate and multivariate logistic regression analysis between the onset of menopausal age and gynecologic cancers are shown in **Table 2**. There was an inverse association between age at menopause and the prevalence of gynecological cancer (OR: 0.93, 95% CI: 0.91,0.95), and the difference was statistically significant (*P*<0.01). Model 1 was adjusted for age and race, and the results showed that there was an inverse association between menopausal age and the prevalence of gynecological cancer (OR: 0.92, 95% CI: 0.90-0.94), and the difference was statistically significant (*P*<0.01). Model 2 was adjusted for age, race, first menstruation age, and living birth, and showed an inverse association between age at menopause and gynecologic cancer (OR: 0.92, 95% CI: 0.90-0.94), with statistically significant differences (*P <*0.01). Model 3 was adjusted for age, race, first menstruation age, living birth, education, BMI, PIR, smoking, alcohol consumption level, energy intake, and showed that there was an inverse association between age at menopause and the prevalence of gynecologic cancer (OR: 0.92, 95% CI: 0.91-0.94), and the difference was statistically significant (*P <*0.01). Model 4 was adjusted for age, race, first menstruation age, living birth, education, BMI, PIR, smoking, alcohol consumption level, energy intake, hypertension, and diabetes, and showed that there was an inverse association between age at menopause and the prevalence of gynecologic cancer (OR: 0.92, 95% CI: 0.90-0.94), and the difference was statistically significant (*P <*0.01). Furthermore, to evaluate the effect of specific factors on the gynecological cancers, we performed subgroup analysis and the results are shown in **Supplementary Table S1**.

**Table 2:** Association between age of menopause and gynecologic cancer.

Outcomes	Model	OR (95% CI)	*P* value
Gynecological Cancer	Crude	0.93 (0.91, 0.95)	<0.01
	Model 1	0.92 (0.90, 0.94)	<0.01
	Model 2	0.92 (0.90, 0.94)	<0.01
	Model 3	0.92 (0.91, 0.94)	<0.01
	Model 4	0.92 (0.90, 0.94)	<0.01

OR, odds ratio; CI, confidence interval. Crude is an unadjusted model; model 1 is a model adjusted for age and race; model 2 is a model adjusted for age, race, first menstruation age and living birth; model 3 is a model adjusted for age, race, first menstruation age, living birth, education, BMI, PIR, smoking, alcohol consumption level, energy intake; Model 4 is a model adjusted for age, race, first menstruation age, living birth, education, BMI, PIR, smoking, alcohol consumption level, energy intake, hypertension and diabetes.

### Relationship between the age of menopause and the prevalence of major gynecological cancers

**Figure 2:**
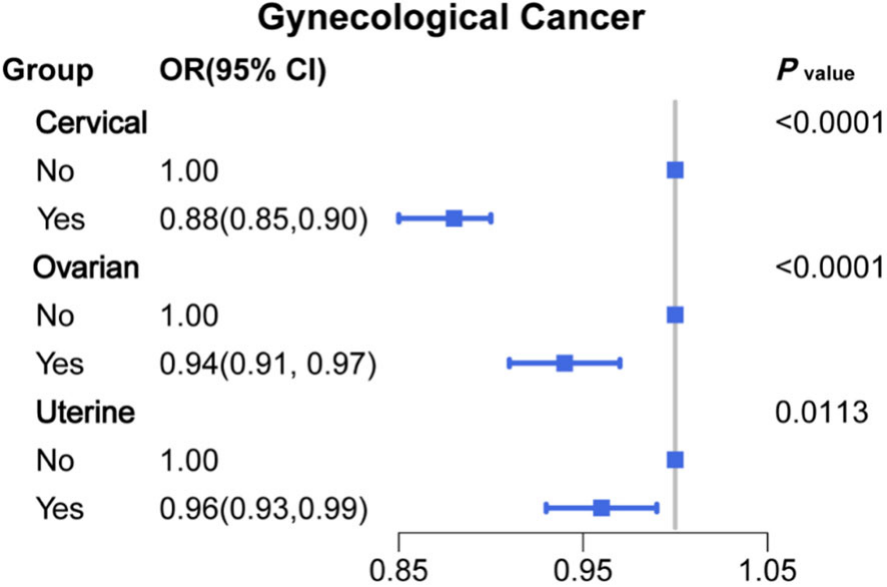
Associations between age at menopause and major gynecological cancer (OR (95% CI) and P value).

**Figure 3:**
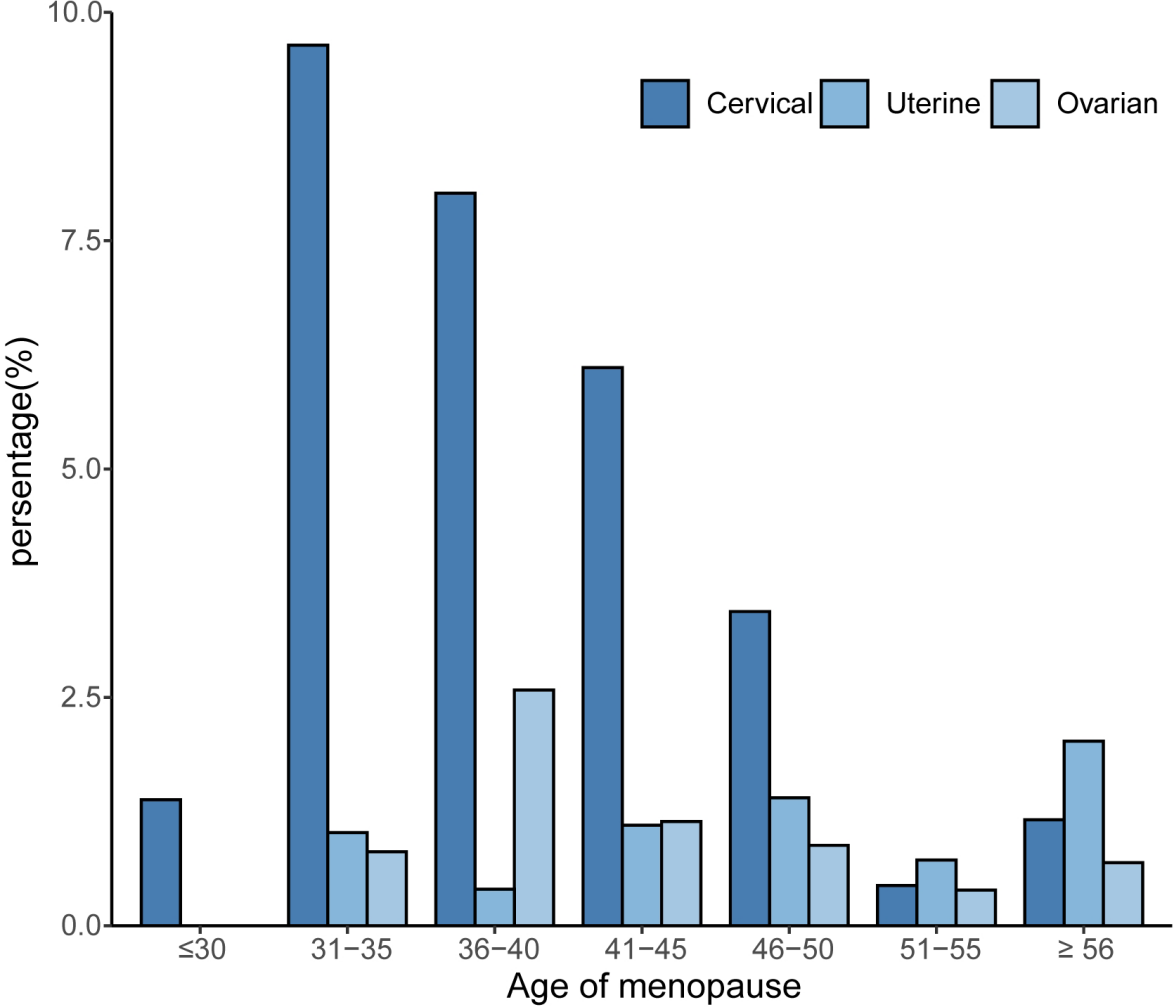
Relationship between age at menopause and the prevalence of gynecological cancer by age group.

To investigate whether menopause is associated with gynecologic age, we performed a regression analysis between menopause and the incidence of three major gynecologic cancers, as shown in **Figure 2** for the relationship between age at menopause and the incidence of different gynecologic cancers. After adjusting for age, race, first menstruation age, living birth, education, BMI, PIR, smoking, alcohol consumption level, energy intake, hypertension, and diabetes (model 4), the regression results revealed that age at menopause was inversely associated with the prevalence of gynecologic cancers in patients with cervical (OR: 0.88, 95% CI: 0.85-0.90), ovarian (OR: 0.94, 95% CI: 0.91-0.97) and uterine cancer (OR: 0.96, 95% CI: 0.93-0.99). According to different menopause ages, participants were divided into 7 groups, as shown in **Figure 3**. The percentage of major gynecological cancers occurring at different menopause age groups were observed respectively. The results showed that participants aged 31-35 years had a higher incidence of cervical cancer than other age groups. Cervical cancer incidence decreased gradually among participants aged 31-45 years, with statistical significance (*P <*0.01). The incidence of uterine cancer was higher in participants aged 56 years or older at menopause than in other age groups. Menopausal participants aged 36 to 40 years had a higher incidence of ovarian cancer than the rest of the age group. To further explore more specific associations, subgroup analysis was performed for cervical cancer, ovarian cancer, and uterine cancer, and the results are shown in **Supplementary Tables S2**–**S4**.

### Nonlinear relationship between age of menopause and the prevalence of gynecological cancers

**Figure 4:**
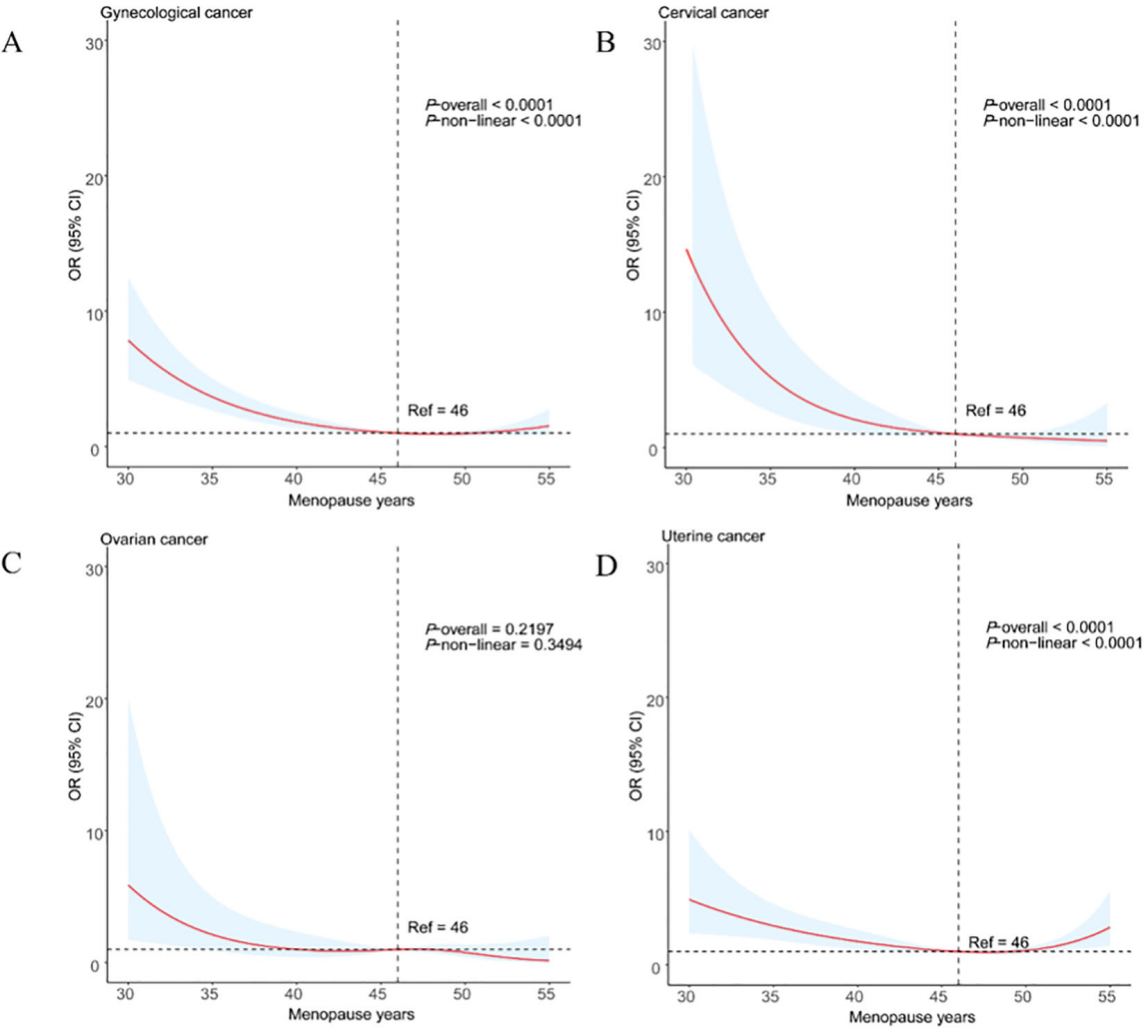
Restricted cubic spline analysis of age at menopause and major gynecological cancer **(A)** gynecological cancer; **(B)** cervical cancer; **(C)** ovarian cancer; **(D)** uterine cancer).

By using the RCS models with full adjustment for confounders, the results showed that there was a low L-shaped association between age at menopause and the prevalence of gynecological cancer (**Figure 4A**). In addition, the results also found that there was a linear association between age at menopause and cervical and uterine cancer (**Figures 4B, D**), but not with ovarian cancer (**Figure 4C**).

## Discussion

This study revealed that women with an earlier age at menopause face a significantly higher risk of gynecologic cancers (cervical, ovarian, and uterine cancers), supporting the inverse relationship between menopause age and cancer risk. The rapid drop in estrogen associated with early menopause is a key factor in the elevated cancer risk. Previous studies indicate that a quick decline in estrogen may impair tissue repair mechanisms for DNA damage, increase apoptosis, and contribute to chronic inflammation, all of which elevate cancer risks (26–28). A study reported the trends in incidence and mortality rates of cervical cancer in China and analyzed the independent effects of age, period, and cohort on these trends. The results showed that the incidence of cervical cancer has increased among young women under the age of 35 (29). Additionally, a study assessed the incidence, disability-adjusted life years (DALYs), and mortality rates of cervical cancer and found that the incidence has increased among younger age groups, especially among women under the age of 35 (30), which is consistent with the results of the 31-35 age group mentioned in this study.

Moreover, subgroup analysis in this study further refined the risk differences associated with different menopause age groups, showing that women who reached menopause between 36-40 years had a significantly higher risk of ovarian cancer, while women who reached menopause after age 56 had an increased risk of uterine cancer. This finding supports the hypothesis in the literature that late menopause may increase the risk of uterine cancer due to prolonged exposure to high estrogen levels, leading to persistent endometrial stimulation and an elevated risk of uterine cancer (15, 31, 32).

When analyzing the relationship between age at menopause and the risk of gynecological cancer, our study employed RCS and found a low L-shaped relationship between age at menopause and the prevalence of gynecological cancer. This finding is consistent with existing literature, particularly in understanding the impact of changes in estrogen levels on the risk of gynecological cancer.

Firstly, a study based on the NHANES database indicated that a univariate logistic regression analysis of age at menopause and the prevalence of gynecological tumors showed a negative correlation between age at menopause and the prevalence of common gynecological tumors. Particularly for ovarian and cervical cancers, after adjusting for the effects of covariates, a higher risk of gynecological tumors was found, and there were statistically significant differences at earlier ages of menopause. This is in line with our research results, suggesting that before a certain critical point, a lower age at menopause significantly increases the risk of gynecological cancer (33). Furthermore, research has shown that women carrying pathogenic BRCA1/2 gene mutations have up to an 87% risk of developing related cancers. Specifically, multiple breast cancer clusters in BRCA1 and BRCA2 are associated with relatively higher risks of breast cancer and relatively lower risks of ovarian cancer. These findings further emphasize the role of genetic factors in the risk of gynecological cancer and how age at menopause may interact with these genetic risk factors (34). In summary, our research results are consistent with existing literature, highlighting the complex relationship between age at menopause, changes in hormone levels, and the risk of gynecological cancer. These findings provide important scientific evidence for future prevention strategies and intervention measures, especially in identifying high-risk groups and developing personalized prevention plans. We observed a linear inverse relationship between menopause age and the incidence of cervical and uterine cancers, while ovarian cancer showed no significant trend, possibly due to its complex etiology and differing sensitivity to hormones (33, 35, 36).

The chronic inflammatory state post-menopause is also considered a key mechanism in the increase. A 4-year follow-up study that explores the relationship between metabolic health, menopause, and physical activity. The study results indicate that menopause and levels of physical activity have a significant impact on the metabolic health of middle-aged women (37). A literature review based on data from the Study of Women’s Health Across the Nation (SWAN), examines the relationship between menopause and metabolic syndrome. The study found that menopause is associated with changes in cardiovascular disease risk factors, which are also related to cancer risk. The study also revealed common genetic signatures associated with metabolic syndrome, type 2 diabetes, cardiovascular diseases, and menopausal status, which are significantly enriched in biological processes, including the positive regulation of binding, the positive regulation of leukocyte cell adhesion, and the regulation of lipid localization (38). Visceral fat accumulation is associated with an increased risk of various cancers, including those of the uterus, cervix, breast, liver, and ovaries. The study also notes that obesity can interfere with therapies and contribute to morbidity from chemotherapy toxicities, thus promoting worse prognosis and mortality (39). The study found that higher levels of insulin resistance are associated with higher breast cancer incidence and higher all-cause mortality after breast cancer (40). Research findings indicate that the link between visceral adipose tissue and cancer risk may involve systemic mechanisms, such as leptin, glucose, insulin, and inflammatory cytokines, which are systemic markers of obesity-related adipose tissue inflammation and may promote tumor development (41). Chronic inflammation may play an important role in the pathogenesis of non-inflammatory diseases such as breast cancer. Activation of innate immunity creates a tissue microenvironment rich in reactive oxygen and nitrogen species that may lead to DNA damage and changes in nearby cells, the study suggests. Inflammation also raises circulating levels of inflammatory cytokines that promote cancer, such as C-reactive protein (CRP) and interleukin-6 (IL-6) (42). There are also studies showing that links between chronic low-grade inflammatory states and multiple chronic diseases are now evident, and controlling this condition may be important to prevent the most common diseases in the general population (43). A case-control study that prospectively assessed whether plasma levels of inflammatory markers such as CRP, TNF-α, IL-6, leptin, and adiponectin were associated with breast cancer risk showed no significant association between these inflammatory markers and breast cancer risk but found significant interactions between menopausal status and plasma levels. All of these studies support the scientific evidence for a relationship between postmenopausal chronic inflammatory state and cancer risk, and support the idea that postmenopausal chronic inflammatory state may be one of the key mechanisms for increased cancer risk (44). This inflammatory state aligns with our findings, supporting the inclusion of early menopausal women in high-risk cancer screening groups.

In addition to the elevated risk for early menopausal women, late menopausal women also face specific health risks. A study used a meta-analysis to evaluate the relationship between unopposed estrogen or estrogen plus progesterone and endometrial cancer risk. The results showed that women who use estrogen have a higher relative risk than non-users. Risk (RR 2.3) was associated with prolonged use (RR 9.5 for 10 years or more), and the risk of endometrial cancer remained elevated even after 5 years or more of discontinuation of unopposed estrogen therapy (RR 2.3) (45). A systematic review assessed the safety of estrogen plus progestin therapy, particularly considering the impact of treatment regimens and types of progestins on the risk of endometrial cancer. The study found that women who used estrogen alone had an increased risk, while continuous combined therapy was associated with a lower risk compared to sequential combined therapy (46). Hormone replacement therapy should be used with caution in women with a higher risk of endometrial cancer (HR 2.84) in those with a later menopause (age ≥55 years) than in those with the youngest menopause (<45 years)15. These studies underscore the importance of menopause age in gynecologic cancer screening and intervention strategies.

Recently, more studies have viewed age at menopause as an outcome of multiple interacting factors, further highlighting its unique impact on cancer risk. A study points out that lifestyle and dietary factors determine the age of natural menopause. The research indicates that a healthy diet and regular exercise are significant factors affecting the age of menopause, thereby potentially indirectly influencing the cancer risks associated with early menopause (47). A systematic review and meta-analysis studied the impact of psychological interventions on the quality of life of early-stage cancer patients. The study included psychological interventions such as cognitive-behavioral therapy, relaxation training, meditation, stress management, and self-help, which are believed to improve patients’ quality of life and may indirectly affect cancer risk (41). There is also a method called “emotional support and case finding” used for the clinical management of cancer patients’ emotions. This approach emphasizes the importance of psychological support in cancer treatment and may help reduce cancer risk (48). Psychological interventions for cancer patients include cognitive-behavioral therapy, art therapy, and relaxation therapy, among others. These interventions aim to improve patients’ emotional states and quality of life, which may positively impact the reduction of cancer risk (49).

This study makes a significant contribution by providing an in-depth analysis of the relationship between age at menopause and the risk of three major gynecological cancers: cervical, ovarian, and endometrial cancer. The results indicate a notable association: early menopause correlates with an increased risk of cervical and ovarian cancers, whereas late menopause is associated with a higher risk of endometrial cancer. These findings provide a scientific foundation for future personalized screening and health intervention strategies. The inverse association between menopausal age and cancer prevalence suggests that early menopause may be a marker for increased risk, prompting more frequent monitoring and targeted screening for women who experience menopause at younger ages. Additionally, the nonlinear relationship observed highlights the need for personalized risk assessments, taking into account individual factors such as age at menopause, lifestyle, and family history, to optimize prevention and early detection strategies for gynecological cancers. Unlike previous research that broadly examined the link between menopausal age and cancer risk, this study categorizes menopausal age into specific age groups and conducts a subgroup analysis across different cancer types, thereby revealing age-specific cancer risks. Furthermore, by employing a multilevel regression model and adjusting for various confounding variables, the study clarifies the independent effect of menopausal age on cancer risk, enhancing the statistical robustness of the findings. Additionally, the use of the large, representative NHANES database lends strong external validity to the study. NHANES data encompass participants from diverse racial, socioeconomic, and health backgrounds, enhancing the generalizability of the findings. Many previous studies, limited by small sample sizes or specific populations, restricted the applicability of their results. By leveraging NHANES’s extensive dataset, this study addresses these limitations and offers a robust reference point for personalized cancer screening in various populations. Another notable achievement of this study is its exploration of a potential nonlinear relationship between menopausal age and gynecological cancer risk. Using RCS regression models, the study is among the first to suggest an L-shaped nonlinear association, indicating that cancer risk may not increase linearly with menopausal age but could be influenced by a combination of factors, with critical risk thresholds for different age groups. This insight provides important theoretical support for age-segmented clinical management strategies.

Despite these valuable insights, the study has several limitations. First, as a retrospective analysis based on cross-sectional data from the NHANES database, it cannot establish causation. Though we have adjusted for multiple confounding factors, the possibility of reverse causation cannot be ruled out. Future longitudinal studies are needed to confirm the causal link between menopausal age and cancer risk, clarifying whether early menopause directly contributes to elevated cancer risk or if other intermediary factors are involved. Second, the study relies on self-reported data, including menopausal age, menarche age, and lifestyle factors, which may introduce recall bias and reporting inaccuracies. Participants may not accurately recall age-related events or health behaviors, particularly over long periods. Future research should incorporate objective biomarkers to reduce self-reporting errors. For example, hormonal and inflammatory biomarkers could more precisely measure physiological changes associated with menopause and their correlation with cancer risk. Moreover, this study does not delve into the variability in the relationship between menopausal age and cancer risk across different demographic groups (e.g., by race, socioeconomic status, and living environment). Both menopausal age and gynecological cancer incidence may vary significantly across racial and socioeconomic groups, especially in terms of lifestyle factors and healthcare access. Future studies should analyze these differences in greater detail to understand how menopausal age distribution and its impact on cancer risk vary across populations, which would aid in developing more targeted and equitable health management strategies, improving the efficiency of cancer screening and prevention. Finally, the study does not fully explore the biological mechanisms underlying the association between menopausal age and cancer risk. Although hypotheses around estrogen decline and chronic inflammation are proposed, these mechanisms require further verification through experimental and longitudinal studies. Future research could employ animal models or clinical trials to investigate how menopause-induced physiological changes specifically contribute to cancer development, thereby offering a biological basis for prevention and therapeutic strategies.

## Conclusion

This study provides significant insights into the association between age at menopause and the risk of developing gynecologic cancers, particularly cervical, ovarian, and uterine cancers. Our findings underscore the role of early menopause as a risk factor for these cancers, while highlighting late menopause as an associated risk for uterine cancer. By employing a large, representative sample and robust analytical methods, our research contributes to the understanding of menopause’s impact on cancer risks. These results have potential implications for clinical practice, suggesting that menopausal age could be a critical factor in developing personalized cancer screening strategies. Future studies, ideally longitudinal in design, are essential to further elucidate the causal pathways involved and to explore the biological mechanisms underlying these associations. Such efforts could pave the way for targeted preventive measures and more effective health interventions for women across different menopausal age groups.

## Data Availability

Publicly available datasets were analyzed in this study. This data can be found here: https://www.cdc.gov/nchs/nhanes/index.html.
