# Supplementary material for: The influence of menopause age on gynecologic cancer risk: a comprehensive analysis using NHANES data
Source: Front Oncol. 2025 Feb 11;15:1541585. doi: 10.3389/fonc.2025.1541585 (PMC11851290; doi:10.3389/fonc.2025.1541585)
Supplement: Supplementary file 1 [file Table1.docx]

Supplementary Material

The Influence of Menopause Age on Gynecologic Cancer Risk: A Comprehensive Analysis Using NHANES Data

**Abulajiang,Yiliminuer**^†^**, Tao Liu**^†^**, Ming Wang, Abulai,Abidan, Yumei Wu***

^†^These authors contributed equally to this work

*** Correspondence:** Yumei Wu, Email: wym597118@ccmu.edu.cn

# Supplementary Figures and Tables

## Supplementary Tables

**Supplementary Table 1: Stratified analysis of gynaecological cancer.**

| **Subgroup Variable** | **Cancer = gynaecological (95%CI)** | ***P* value** |
| --- | --- | --- |
| Age |  |  |
| < 65 | 0.91 (0.88, 0.93) | **<0.0001** |
| ≥ 65 | 0.97 (0.94, 1.01) | 0.1023 |
| Race |  |  |
| Non-Hispanic White | 0.93 (0.91, 0.95) | **<0.0001** |
| Non-Hispanic Black | 0.96 (0.92, 1.00) | **0.0380** |
| Mexican American | 0.93 (0.88, 0.98) | **0.0110** |
| Other Hispanic | 0.94 (0.89, 1.00) | 0.0600 |
| Other Race | 0.88 (0.83, 0.95) | **0.0005** |
| Family PIR |  |  |
| < 2.3 | 0.92 (0.90, 0.94) | **<0.0001** |
| ≥ 2.3 | 0.94 (0.91, 0.97) | **0.0005** |
| Education level |  |  |
| Less than high school | 0.90 (0.87, 0.93) | **<0.0001** |
| High school or equilent | 0.94 (0.90, 0.98) | **0.0095** |
| College or above | 0.93 (0.91, 0.96) | **<0.0001** |
| BMI |  |  |
| < 25 | 0.92 (0.89, 0.95) | **<0.0001** |
| ≥ 25 | 0.93 (0.91, 0.96) | **<0.0001** |
| Smoke behavior |  |  |
| Never | 0.94 (0.92, 0.97) | **<0.0001** |
| Former | 0.91 (0.88, 0.94) | **<0.0001** |
| Now | 0.92 (0.88, 0.96) | **<0.001** |
| Alcohol comsumption |  |  |
| Never | 0.97 (0.94, 1.01) | 0.2112 |
| Former | 0.91 (0.87, 0.94) | **<0.0001** |
| Mild | 0.95 (0.92, 0.98) | **0.0006** |
| Moderate | 0.91 (0.87, 0.95) | **<0.0001** |
| Heavy | 0.92 (0.84, 1.00) | 0.0647 |
| Energy intake |  |  |
| < 1580 | 0.92 (0.89, 0.95) | **<0.0001** |
| ≥ 1580 | 0.94 (0.91, 0.96) | **<0.0001** |
| Hypertension |  |  |
| Yes | 0.94 (0.90, 0.98) | **0.0018** |
| No | 0.93 (0.90, 0.95) | **<0.0001** |
| Diabetes |  |  |
| Yes | 0.93 (0.90, 0.97) | **0.0004** |
| No | 0.93 (0.91, 0.95) | **<0.0001** |

Note: Bold indicates *P* value < 0.05.

**Supplementary Table 2: Stratified analysis of cervical cancer.**

| **Subgroup Variable** | **Cancer = cervical (95%CI)** | ***P* value** |
| --- | --- | --- |
| Age |  |  |
| < 65 | 0.87 (0.84, 0.90) | **<0.0001** |
| ≥ 65 | 0.92 (0.87, 0.98) | **0.0068** |
| Race |  |  |
| Non-Hispanic White | 0.88 (0.85, 0.91) | **<0.0001** |
| Non-Hispanic Black | 0.93 (0.87, 1.00) | 0.0526 |
| Mexican American | 0.88 (0.83, 0.93) | **<0.0001** |
| Other Hispanic | 0.00 (0.00, 0.00) | **<0.0001** |
| Other Race | 0.78 (0.70, 0.86) | **<0.0001** |
| Family PIR |  |  |
| < 2.3 | 0.89 (0.86, 0.91) | **<0.0001** |
| ≥ 2.3 | 0.87 (0.84, 0.91) | **<0.0001** |
| Education level |  |  |
| Less than high school | 0.88 (0.84, 0.93) | **<0.0001** |
| High school or equilent | 0.88 (0.85, 0.91) | **<0.0001** |
| College or above | 0.88 (0.85, 0.91) | **<0.0001** |
| BMI |  |  |
| < 25 | 0.88 (0.84, 0.93) | **<0.0001** |
| ≥ 25 | 0.88 (0.85, 0.90) | **<0.0001** |
| Smoke behavior |  |  |
| Never | 0.89 (0.85, 0.93) | **<0.0001** |
| Former | 0.84 (0.80, 0.88) | **<0.0001** |
| Now | 0.90 (0.87, 0.93) | **<0.0001** |
| Alcohol comsumption |  |  |
| Never | 0.97 (0.87, 1.07) | 0.5150 |
| Former | 0.86 (0.81, 0.92) | **<0.0001** |
| Mild | 0.87 (0.83, 0.91) | **<0.0001** |
| Moderate | 0.86 (0.80, 0.92) | **<0.0001** |
| Heavy | 0.83 (0.77, 0.89) | **<0.0001** |
| Energy intake |  |  |
| < 1580 | 0.89 (0.85, 0.93) | **<0.0001** |
| ≥ 1580 | 0.87 (0.85, 0.90) | **<0.0001** |
| Hypertension |  |  |
| Yes | 0.91 (0.85, 0.98) | **0.0133** |
| No | 0.87 (0.84, 0.89) | **<0.0001** |
| Diabetes |  |  |
| Yes | 0.90 (0.83, 0.96) | **0.0034** |
| No | 0.88 (0.85, 0.90) | **<0.0001** |

Note: Bold indicates *P* value < 0.05.

**Supplementary Table 3: Stratified analysis of ovarian cancer.**

| **Subgroup Variable** | **Cancer = Ovarian (95%CI)** | ***P* value** |
| --- | --- | --- |
| Age |  |  |
| < 65 | 0.92 (0.88, 0.95) | **<0.0001** |
| ≥ 65 | 0.99 (0.93, 1.04) | 0.6501 |
| Race |  |  |
| Non-Hispanic White | 0.95 (0.92, 0.98) | **0.0032** |
| Non-Hispanic Black | 0.95 (0.88, 1.02) | 0.1577 |
| Mexican American | 1.01 (0.94, 1.09) | 0.7849 |
| Other Hispanic | 0.84 (0.76, 0.92) | **0.0005** |
| Other Race | 0.73 (0.60, 0.89) | **0.0021** |
| Family PIR |  |  |
| < 2.3 | 0.91 (0.88, 0.95) | **<0.0001** |
| ≥ 2.3 | 0.97 (0.93, 1.01) | 0.0993 |
| Education level |  |  |
| Less than high school | 0.90 (0.86, 0.93) | **<0.0001** |
| High school or equilent | 0.95 (0.90, 1.00) | **0.0375** |
| College or above | 0.96 (0.90, 1.02) | 0.1575 |
| BMI |  |  |
| < 25 | 1.03 (0.92, 1.15) | 0.5970 |
| ≥ 25 | 0.92 (0.90, 0.95) | **<0.0001** |
| Smoke behavior |  |  |
| Never | 0.95 (0.91, 1.00) | **0.0488** |
| Former | 0.94 (0.89, 1.00) | **0.0445** |
| Now | 0.87 (0.83, 0.91) | **<0.0001** |
| Alcohol comsumption |  |  |
| Never | 1.01 (0.94, 1.09) | 0.7706 |
| Former | 0.91 (0.86, 0.97) | **0.0022** |
| Mild | 0.94 (0.89, 1.00) | **0.0466** |
| Moderate | 0.97 (0.90, 1.04) | 0.3758 |
| Heavy | 0.87 (0.67, 1.12) | 0.2831 |
| Energy intake |  |  |
| < 1580 | 0.93 (0.88, 0.98) | **0.0048** |
| ≥ 1580 | 0.96 (0.92, 0.99) | **0.0211** |
| Hypertension |  |  |
| Yes | 0.91 (0.86, 0.96) | **0.0011** |
| No | 0.95 (0.92, 0.98) | **0.0043** |
| Diabetes |  |  |
| Yes | 0.88 (0.82, 0.94) | **<0.001** |
| No | 0.96 (0.92, 0.99) | **0.0118** |

Note: Bold indicates *P* value < 0.05.

**Supplementary Table 4: Stratified analysis of uterine cancer.**

| **Subgroup Variable** | **Cancer = Uterine (95%CI)** | ***P* value** |
| --- | --- | --- |
| Age |  |  |
| < 65 | 0.96 (0.91, 1.01) | 0.1459 |
| ≥ 65 | 0.98 (0.94, 1.03) | 0.4586 |
| Race |  |  |
| Non-Hispanic White | 0.97 (0.93, 1.01) | 0.1982 |
| Non-Hispanic Black | 0.99 (0.93, 1.05) | 0.7375 |
| Mexican American | 0.98 (0.89, 1.09) | 0.7411 |
| Other Hispanic | 0.97 (0.93, 1.02) | 0.1975 |
| Other Race | 0.92 (0.83, 1.01) | 0.0792 |
| Family PIR |  |  |
| < 2.3 | 0.95 (0.93, 0.98) | **<0.0001** |
| ≥ 2.3 | 0.98 (0.93, 1.05) | 0.6083 |
| Education level |  |  |
| Less than high school | 0.92 (0.88, 0.96) | **0.0004** |
| High school or equilent | 0.97 (0.91, 1.04) | 0.3912 |
| College or above | 1.00 (0.96, 1.04) | 0.9697 |
| BMI |  |  |
| < 25 | 0.93 (0.89, 0.97) | **<0.001** |
| ≥ 25 | 0.98 (0.94, 1.03) | 0.4479 |
| Smoke behavior |  |  |
| Never | 0.96 (0.92, 1.00) | 0.0672 |
| Former | 0.97 (0.93, 1.02) | 0.2569 |
| Now | 0.97 (0.90, 1.05) | 0.4634 |
| Alcohol comsumption |  |  |
| Never | 0.97 (0.92, 1.02) | 0.2008 |
| Former | 0.93 (0.89, 0.98) | **0.0036** |
| Mild | 1.02 (0.97, 1.07) | 0.4642 |
| Moderate | 0.97 (0.94, 1.02) | 0.2232 |
| Heavy | 0.96 (0.89, 1.04) | 0.2920 |
| Energy intake |  |  |
| < 1580 | 0.94 (0.90, 0.98) | **0.0078** |
| ≥ 1580 | 1.00 (0.95, 1.05) | 0.9823 |
| Hypertension |  |  |
| Yes | 0.94 (0.89, 1.00) | **0.0471** |
| No | 0.98 (0.94, 1.03) | 0.5002 |
| Diabetes |  |  |
| Yes | 0.95 (0.91, 1.00) | 0.0659 |
| No | 0.97 (0.93, 1.02) | 0.2337 |

Note: Bold indicates *P* value < 0.05.
